# Supplementary material for: Effects of Long-Term Use of Organic Fertilizer with Different Dosages on Soil Improvement, Nitrogen Transformation, Tea Yield and Quality in Acidified Tea Plantations
Source: Plants (Basel). 2022 Dec 26;12(1):122. doi: 10.3390/plants12010122 (PMC9824488; doi:10.3390/plants12010122)
Supplement: Supplementary file 1 [file plants-12-00122-s001.zip › plants-2005565-supplementary.pdf]

Supplementary Materials

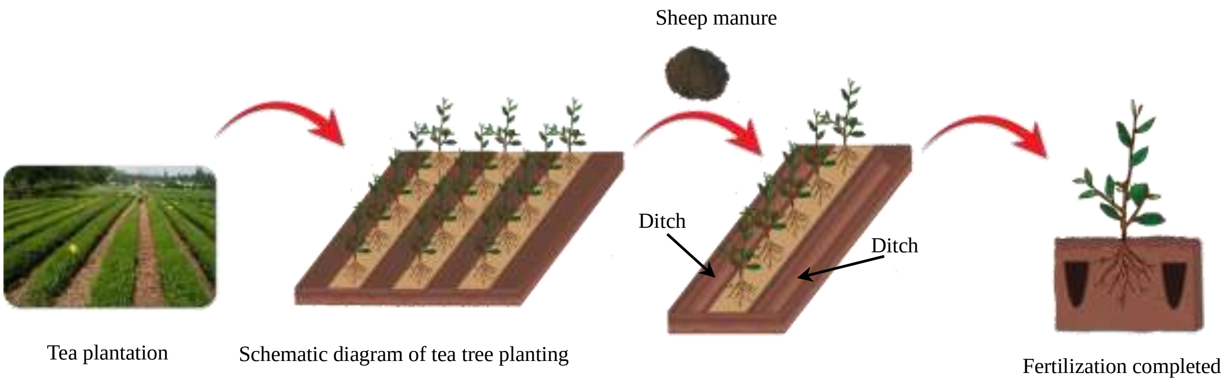

Figure S1 Schematic diagram of how to use sheep manure

Table S1 The tea tree and soil index values of the tea plantation in the experimental site were determined in May 2017

	Sample number	Numerical range	Average	Standard deviation	Coefficient of variation (%)
pH value	175	3.34~3.41	3.38	0.06	1.78
Yield (kg/hm^2)	175	3045~3136	3104	105	3.38
Tea polyphenols (mg/g)	175	148.35~154.26	153.24	3.98	2.60
Theanine (mg/g)	175	5.18~5.41	5.32	0.19	3.57
Amino acid (mg/g)	175	14.32~15.31	14.98	0.37	2.47
Caffeine (mg/g)	175	13.25~14.31	13.86	0.41	2.96
Nitrate nitrogen (mg/kg)	175	63.94~66.08	65.32	1.29	1.97
Ammonium nitrogen (mg/kg)	175	0.65~1.38	1.03	0.24	23.30
Nitrifying bacteria(10^5 cfu/g·soil)	175	18.87~21.45	20.36	0.29	1.42
Ammonifying bacteria(10^7 cfu/g·soil)	175	7.98~8.52	8.37	0.18	2.15
Nitrification intensity (%)	175	33.53~35.06	34.15	0.42	1.23
Ammoniation strength (mg/100 mL)	175	16.89~19.43	18.79	1.08	5.75
Urease (μmol/min·L)	175	138.74~144.23	141.28	2.62	1.85
Protease (μmol/min·L)	175	7.85~9.03	8.57	0.57	6.65
Nitrate reductase (μmol/min·L)	175	6.81~7.34	7.13	0.26	3.65
Nitrite reductase (μmol/min·L)	175	168.52~175.91	173.25	3.89	2.25
